# Assessment of Anxiety, Depression, Stress, and Associated Psychological Morbidities among Patients Receiving Ayurvedic Treatment for Different Health Issues: First Study from Sri Lanka

**DOI:** 10.1155/2019/2940836

**Published:** 2019-11-28

**Authors:** Hiruni Jayagrahi Gunathilaka, Pemasiri Vitharana, Lahiru Udayanga, Nayana Gunathilaka

**Affiliations:** ^1^Gampaha Wickramarachchi Ayurveda Institute, University of Kelaniya, Yakkala, Sri Lanka; ^2^Department of Bio-Systems Engineering, Faculty of Agriculture and Plantation Management, Wayamba University, Makandura, Sri Lanka; ^3^Department of Parasitology, Faculty of Medicine, University of Kelaniya, Ragama, Sri Lanka

## Abstract

**Background:**

Good mental condition is a vital part of health. Physical impairments would potentially have psychiatric manifestations during the course of a disease that could cause patients to experience a wide range of psychological conditions. This study was conducted to determine prevalence of anxiety, depression, stress, and psychological morbidities among the patients who received warded treatments at Gampaha Wickramarachchi Ayurveda Teaching Hospital, Sri Lanka.

**Methods:**

A total of 148 patients admitted to the hospital were selected for the study on a random systematic basis under four systemic groups (gastrointestinal, integumentary, musculoskeletal, and nervous system) depending on the chief complaint. The presence of depressive, anxiety, and stress symptoms was assessed by the Depression Anxiety Stress Scale 21 item version (DASS 21). The General Linear Model (GLM) was used for statistical analysis.

**Results:**

Over 50% of the participants in all four patient groups belonged to age group of 35 to 65 years, encompassing the fraction of population that actively contribute to the workforce in the society. Stress, anxiety, and depression values of patients belonging to different complications varied significantly, as indicated by GLM (*p* < 0.05). Patients diagnosed with integumentary system-related issues denoted the highest stress levels (27.7 ± 2.54), while the mean stress values among the other systemic groups were not significantly different among each other. The highest anxiety levels were indicated by patients with nervous system-related issues (18.6 ± 1.51), while the lowest anxiety levels were indicated by patients with integumentary disorders (6.0 ± 2.73). The highest depression level was identified from patients suffering from integumentary system-related disorders (31.7 ± 3.42), followed by nervous system (23.2 ± 1.78), gastrointestinal (19.5 ± 3.77), and musculoskeletal (16.8 ± 1.57) disorders.

**Conclusion:**

Overall, high distress levels were observed among the majority of the patients. Furthermore, integumentary issues may lead to significant psychological impacts. As most of the patients seek for Ayurveda treatments when their diseased condition becomes chronic, it is vital to focus on a biopsychosocial approach to patient assessment and patient care, in actual practice.

## 1. Introduction

A good health condition in a living being is an important aspect in their life. According to the World Health Organization (WHO), health is defined as “a state of complete physical, mental and social well-being and not merely the absence of disease or infirmity” [1]. Even though the majority of the patient's chief complaints are not related to psychological morbidities, there is experimental evidence that they have some psychological problems as well.

Although they have psychological morbidities, they may not receive specific psychiatric treatments. It is well known that physical condition of individuals greatly affects their psychological health [2] and, therefore, psychological health may also influence the physical conditions which may delay the recovery of patients suffering from different physical health issues. Therefore, a better knowledge on the psychological morbidity of the physically diseased would support the planning of a multidisciplinary approach to the care of patients with different physiological disorders in acute, intermediate, and long terms.

According to Ayurveda, simple freedom from diseases is not enough to call a person healthy. In addition, a person should have perfect functioning of the senses, happiness of the mind, and spiritual elevation to be called a “healthy person” in the real sense of the term. As a result of scientific advancement, man has become free from many infectious diseases, and the average life span has been increased. However, that patients suffer from prolonged health issues causes mental unhappiness and leads them to suffer from many invalidating diseases. Therefore, physical impairments would potentially have psychiatric manifestations during the course of a disease, making patients experience a wide range of psychological conditions [3]. This is the primary objective of the prescriptions and prohibitions described in Ayurveda for the preservation and promotion of positive health and prevention as well as cure of diseases.

In Sri Lanka, there is an ancient history of Ayurveda treatment for many health-related complications. With the evolution of Western medicine, people have become prone to obtain Western medicine prescriptions because of its convenience, since Ayurveda treatment needs preparations of drugs and is not readily available as products until recently. However, with the advancement in recent years, many of the Ayurveda treatment drugs are readily available as capsules, tablets, syrup, oil, and powders, which have enormously contributed people to drift towards Ayurveda treatments. On the other hand, the development in the government hospital network for Ayurveda treatment in the country, which is maintained as a free service to the general public, and continuous enrolment of qualified physicians to the government health service from graduates passing out from state universities have also contributed greatly to promote this treatment regimen among general public.

The majority of the patient's chief complaints are not related to psychological morbidities, but while talking to them, it is obvious that they have some psychological problems as well. Although they have psychological morbidities, they may not receive specific psychiatric treatments. Therefore, their progress or recovery rate may be lower. However, physical condition of individuals may greatly influence their psychological health [2]. In some cases, poor psychological conditions of patients may adversely affect the level of recovery and the time taken for curing of physical disorders [2]. Therefore, a better knowledge on the psychological morbidity of the disease would support planning of more effective multidisciplinary caring approaches for patients with different physiological disorders in acute, intermediate, and long terms. This combined approach of patient management has never been used in Sri Lanka, and no published studies are available at Ayurveda setup on psychological status of patients receiving Ayurvedic treatment, which ultimately affect the recovery rate of patients suffering from different illnesses.

In general, most of the patients seek Ayurveda treatments when their disease becomes chronic, by which they are mentally exhausted and their progress rate may be lower. Therefore, it is essential for the physician to adopt a biopsychosocial approach. Although psychiatrists highlight the necessity of adopting a biopsychosocial approach to patient assessment and patient care, in actual practice, they do not inevitably operate within the frame work of such a model. These aspects have not been investigated from patients who receive Ayurveda treatment in Sri Lanka as well as in other countries, where patients are under the care of a traditional or indigenous medicine practitioner. Therefore, the current study focused on determining the anxiety, depression, stress, and psychological morbidities among the patients receiving Ayurveda treatment for different health complaints in Sri Lanka as the first ever study. Hence, the study of this nature will be useful in enhancing the treatment and patient care with psychotherapy at the Ayurveda setup.

## 2. Method

### 2.1. Study Design

A descriptive cross-sectional cohort study design was used. A total of 148 patients suffering from diseases affecting the nervous system (*n*=50), musculoskeletal system (*n*=40), integumentary system (*n*=30), and gastrointestinal system (*n*=28), who were admitted to Gampaha Wickramarachchi Ayurveda Teaching Hospital, Sri Lanka, from August 2017 to January 2018 were randomly selected for the study. The Krejcie and Morgan formula for sample size calculation was used to calculate the sample size of the above four complications, with a population proportion of 0.5 and margin of error of 3.5% at 95% of confidence level [4]. Age- and gender-matched individuals, reported to the outpatient clinic who were not suffering from any considerable mental or physical disorder of the above categories, were randomly selected as the control group (*n*=50). Those who were under 18 years of age or with known mental illness were excluded. The clinical presentations relevant to each complaint were defined as follows.

#### 2.1.1. Nervous System Disorders

These are complaints in the central and peripheral nervous system including brain, spinal cord, cranial nerves, peripheral nerves, nerve roots, autonomic nervous system, neuromuscular junction, and muscles.

#### 2.1.2. Musculoskeletal Disorders

These are the injuries or pain in the human musculoskeletal system including the joints, ligaments, muscles, nerves, tendons, and structures that support limbs, neck, and back.

#### 2.1.3. Integumentary Disorders

These are a variety of diseases, disorders, and injuries. These range from annoying but relatively benign bacterial or fungal infections that are categorized as disorders.

#### 2.1.4. Gastrointestinal Disorders

These are any condition or disease that occurs within the gastrointestinal tract including mouth, esophagus, stomach, small intestine, large intestine, and anus.

### 2.2. Collection of Sociodemographic Data and Medical Records

The sociodemographic and medical history information of the patients was obtained via an interviewer administered structured questionnaire. Specific variables including age, gender, locality, marital status, family status, occupation, education level, social status, monthly income, treatment history, and presence of diagnosed medical disorder were considered. Written consent was obtained from the study participants prior to the interviews, and all data were kept confidential.

### 2.3. Assessment of Depression, Anxiety, and Stress Symptoms among the Patients

The presence of depressive, anxiety, and stress symptoms was assessed by the Depression Anxiety Stress Scale 21 item version (DASS 21) [5]. Validated Sinhalese and Tamil versions were used with the English version where necessary [6–8]. In each participant, the cut-off mark relevant for our population was considered in all three scales. All participants with a score higher than the recommended cut-off mark in either of the subscales or scales were assessed and be referred to the psychiatry unit to be assessed further.

### 2.4. Analysis of Data

The collected data were entered using the Social Sciences (SPSS) version 16.0 software package. The data are kept confidential. Collected data were analyzed using the SPSS version 16.0 software package. Descriptive statistics and frequencies were calculated for each assessed parameter. The individual scores for each subscale and full scale were calculated. General Linear Model followed by Turkey's pairwise comparison was utilized to investigate the significance in the variations in the stress, anxiety, and depression scores among different patient and control populations at 5% level of significance.

The stress, anxiety, and depression scores belonging to each complication category were subjected to a cluster analysis based on Euclidean distance. Further, the Analysis of Similarities (ANOSIM) was utilized to verify the overall clustering status of different complication categories. Thereafter, Distance-Based Redundancy Analysis (dbRDA) was used to identify the underlying segregation patterns of the patients with different complications based on stress, anxiety, and depression levels. The Plymouth Routines in Multivariate Ecological Research version 6 (PRIMER 6) was used for the statistical analysis.

## 3. Results

### 3.1. Differences of Demographic and Socioeconomic Factors among Study Populations

Over 50% of the participants in all four patient groups belonged to the age group of 35 to 65 years. The percentages of patients in different age categories varied significantly, among four patient populations (*p* < 0.05 at 95% level of confidence). It was noted that the females were representing the majority of all patient populations, except for the patients diagnosed with neurological issues (40%), suggesting that females have higher probability for musculoskeletal, integumentary, and gastrointestinal issues than the males (*p* < 0.05 at 95% level of confidence). Higher proportion of patients of the above health issues were residing in semiurban areas, except for patients with integumentary system-related issues that were predominantly observed among patients living in urban settings (43.4%).

The majority of patients observed in the present study were unemployed, except in the study group having gastrointestinal issues, which represented government servants as the susceptible employment category (28.6%). Apart from the patients with neurological issues, patients that had completed only up to primary educations with “Medium” social status were indicated higher representation followed by those with secondary education (50%) and a relatively lower social status (60%) as shown in Table 1.

### 3.2. Grading of Depression Anxiety Stress Scale 21-Item Version (DASS 21)

Significant differences among calculated DASS scores were noted among the control and patient populations (Table 2). The age- and gender-matched control group indicated the lowest mean stress, anxiety, and depression scores, which were statistically significant from the other patient populations (*p* < 0.05 at 5% level of significance). Patients diagnosed with integumentary system-related issues denoted the highest stress levels, while the mean stress values among the patient populations were not significantly different among each other. However, the stress scores of the patients were significantly higher than those of the control group (Table 2).

The highest anxiety levels were indicated by the patients with nervous system-related issues while the lowest anxiety levels were indicated by the patients with integumentary disorders. Meanwhile, the patients with integumentary problems had the highest scores for depression also, suggesting that integumentary issues may lead to significant psychological impacts as interpreted by the results of General Linear Model.

### 3.3. Percentage Occurrence of Patients with Different Psychological Morbidities

#### 3.3.1. Depression Levels among Patients with Different Disorders

**Figure 1:**
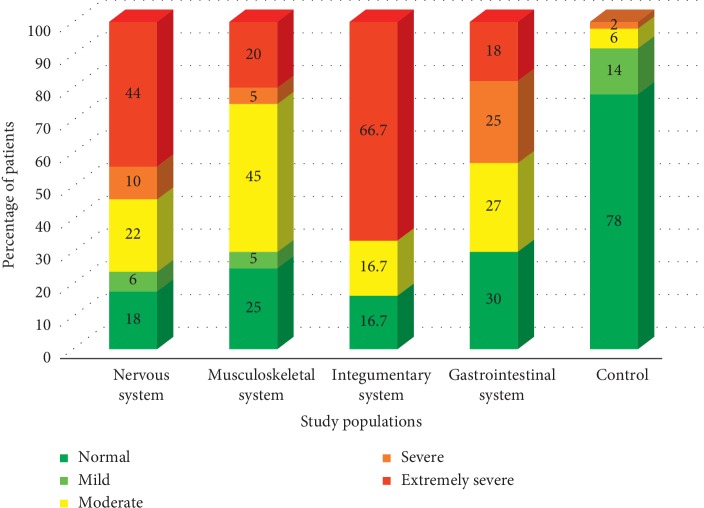
Variation of the percentage of patients belongs to different depression categories.

A high percentage of patients diagnosed with neurological and integumentary-related issues denoted extremely severe levels of depression, while the majority of patients with musculoskeletal issues had moderate depression levels. Interestingly, even though a notable fraction of the patients with gastrointestinal problems were having moderate to extremely severe depression levels, the highest number of patients (30%) were having normal depression scales (Figure 1).

#### 3.3.2. Anxiety Levels among Patients with Different Disorders

**Figure 2:**
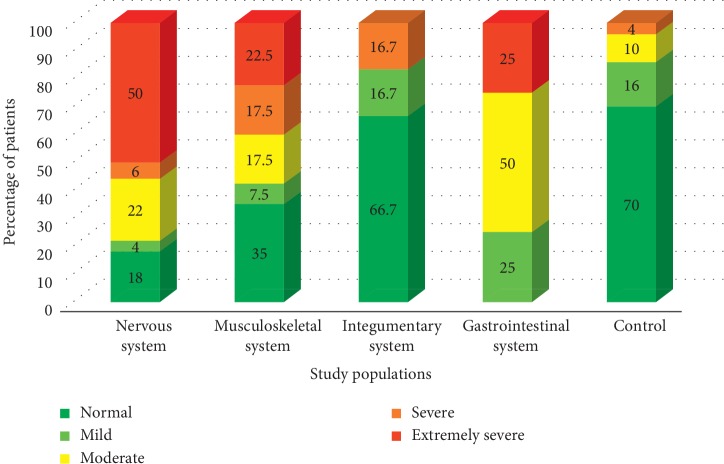
Variation of the percentage of patients belongs to different anxiety categories.

In case of anxiety, extremely severe levels were indicated by 50% of the patients with neurological cases. Surprisingly, most of the patients with integumentary (66.7%) issues were having normal anxiety levels, similar to the control population (70%). However, 57.5% of the patients with musculoskeletal issues and 75% of gastrointestinal issued patients had moderate to extremely severe anxiety levels (Figure 2).

#### 3.3.3. Stress Levels among Patients with Different Disorders

**Figure 3:**
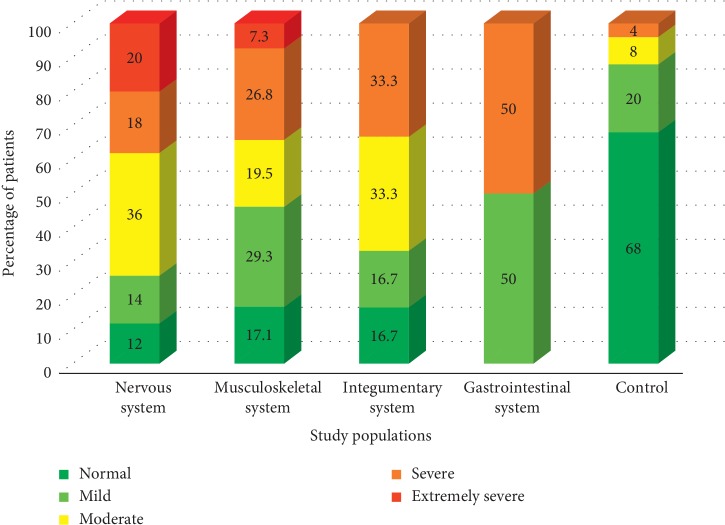
Variation of the percentage of patients belongs to different levels of stress.

Among all the study populations, patients with gastrointestinal issues represented the highest percentage of 50% of patients having severe to extremely severe stress levels, while only one-third (over 33.3%) of other patient groups were falling into the above stress levels. In general, the majority of the patients were characterized by mild or moderate levels of stress in all the study groups except for the control, within which the respondents under the normal stress level (68%) dominated (Figure 3).

### 3.4. Overall Appearance of Patient Populations according to DASS

**Figure 4:**
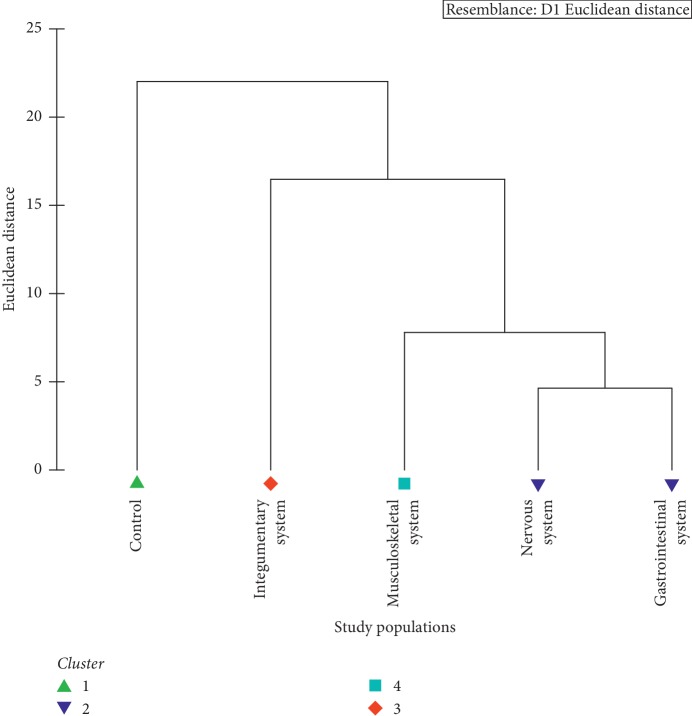
Dendrogram showing the clustering of study populations based on the overall effect of depression, anxiety, and stress.

As suggested by the Euclidean distance-based cluster analysis, patients with neurological and gastrointestinal issues formulate a cluster sharing a notable similarity. Meanwhile, the remaining populations, namely, control and patients with musculoskeletal system-related issues and integumentary system based issues, remain as separate clusters sharing distinct dissimilarities. Based on the overall appearance in terms of depression, anxiety, and stress, four major clusters could be identified among the five study populations (Figure 4).

**Figure 5:**
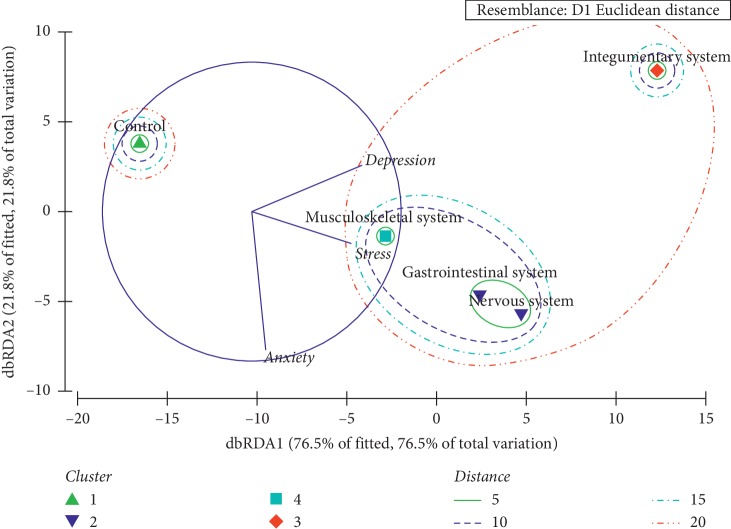
dbRDA plot depicting the clustering of study populations based on the overall effect of depression, anxiety, and stress.

The findings of the Distance-Based Redundancy Analysis (dbRDA) further evidenced the emergence of four major clusters. As suggested by the radiating axes of the dbRDA plot, the patient clusters of musculoskeletal system-related issues remain isolated mainly due to the stress levels, while neurological and gastrointestinal issues populations remain separated due to the interplay of stress and anxiety (Figure 5). Interestingly, the patients having integumentary system-based problems remain as a separate cluster mainly due to the depression levels, whereas the control group remain isolated.

## 4. Discussion

The physical condition of individuals significantly affects their psychological health [8]. Sri Lanka is a developing South-Asian nation with limited mental health resources. The psychiatrist to population ratio has been low compared to the Western countries. However, the number of psychiatrists has increased recently after the existing of civil war in the country for the last 30 years [9]. The mental health literacy among general population appears to be low and the main psychiatric disorders such as schizophrenia were presented to the services after a significantly long duration of untreated psychosis [10].

People living with mental illnesses are at greater risk of experiencing a wide range of physical health problems. The reverse relationship is also true as people living with chronic physical health conditions experience depression and anxiety at twice the rate of the general population [11]. Therefore, it has been well established that psychiatric disorders are quite common among patients with physical illnesses [12]. Psychiatric complications ascending during the course of a general medical condition provide an interface and opportunity to study the organic basis of psychiatric disorders [13]. It is also known that the poor psychological condition of people suffering from disorders ultimately resulted in delaying the recovery and quality of life.

There are only limited studies conducted in Sri Lanka on the grounds of psychological morbidities among different patient groups receiving treatment. Unfortunately, there is no published study available up till now to determine the psychological-related issues among patients who receive Ayurveda and indigenous medicine-based treatments. The present study revealed the first ever attempt to determine the prevalence of anxiety, depression, stress, and psychological morbidities among the patients having complications in nervous system, musculoskeletal system, integumentary system, and gastrointestinal system who received in-house Ayurvedic treatment.

The majority of patient groups were in the age between 35 and 65, which is considered to be the active working force of the country. Therefore, this situation may result in depreciating the quality of life, satisfaction, and further the economic development of the country. Hence, more attention should be drawn to recover these patients physically as well as psychologically. In addition, as many of the patients were females, this will greatly affect the households since household management in Sri Lankan setting is mainly contributed by females.

Psychiatric problems may play a role in the occurrence or relapse of dermatologic disorders; however, it is possible that psychiatric morbidity is secondary to dermatologic disorders as a result of their chronic course, effect on body image, and stigmatic characteristics [14]. According to previous studies, the main psychiatric disorders that appear in dermatology patients were anxiety, depression, and body dysmorphic disorder. AlGhamdi reported that more than half of vitiligo patients describe themselves as either being stressed or depressed [15]. On the other hand, many studies have indicated that the proportion of mood stress and depression among dermatology patients varies according to the location or setting of the study, diagnosis of patients, and tool used to assess the mental illness [16].

The present study revealed that, among the disorders investigated, patients with integumentary system-related issues denoted the highest stress and extremely severe depression levels. Surprisingly, most of the patients with integumentary (66.7%) issues were having normal anxiety levels compared with the control population (70%). Therefore, the current study agrees with the findings of previous published studies conducted in other countries. Hence, the present study also evidenced that the integumentary issues could be headed to significant psychological impacts to the patients.

The symptoms of a nervous system problem depend on which area of the nervous system is involved and what causes the issue. Nervous system-related issues may transpire slowly and cause a gradual loss of function or occur suddenly and cause life-threatening problems. Symptoms may be mild or severe. Therefore, these conditions may be associated with more psychologically related morbidities among patients. According to the present study, patients with nervous system-related issues denoted the highest anxiety levels. The present study also revealed that a higher percentage of patients diagnosed with neurological-related issues indicated extremely severe levels of depression. In case of anxiety, extremely severe levels were indicated by 50% of the patients with neurological cases.

Gastrointestinal disorder is one of the commonest digestive disease entities, which is characterized by frequent gastrointestinal symptoms with no identifiable organic pathology. Some early studies have defined a biopsychosocial pathophysiological model for the pathogenesis of gastrointestinal disorders highlighting the role of biological, social, environmental, and psychological factors for such conditions [17]. However, very few primary care studies have investigated the relationship between gastrointestinal symptoms, depression, and anxiety since depression and anxiety disorders associated with gastrointestinal symptoms commonly occur together [18]. Previous studies conducted on this aspect have denoted higher prevalence of anxiety and depressive symptoms [19–21].

A new classification of GI disorders has accepted that although pathophysiology is not completely implicit, resulting from a complex reciprocal interaction between biological, psychological, and social factors that can be predisposing, precipitating, and/or perpetuating, further, comorbidity with psychiatric disorders, especially mood and anxiety disorders, is high with GI disorders [22]. A study has confirmed that there is a strong association between psychological disorders with GI-related complications and sufficient evidence to support the causative relationship with biological plausibility [19]. Structured clinical interview conducted using the gold standard diagnostic method with psychiatrist and conducted for diagnostic statistical manual of mental disorders-IV axis I disorders has indicated that anxiety disorders diagnosed among considerable number of patients had functional dyspepsia compared with the general population [22–24].

The present study indicated that even though a notable fraction of the patients with gastrointestinal problems were having moderate to extremely severe depression levels, the highest number of patients (30%) were having normal depression scales. Among all study populations, patients with gastrointestinal issues represented the highest percentage of patients having severe to extremely severe stress levels, while only one-third (over 33.3%) of the other patient groups were falling into the above stress levels. About 75% of gastrointestinal issued patients had moderate to extremely severe anxiety levels.

It is important to highlight that the majority of patients who suffered from gastrointestinal-related disorders were employed individuals in the government sector. This may be due to the fact that limited time and care due to their busy routine hinder them to obtain a balance diet, especially at the correct time. On the other hand, psychological stress may also result in aggravating the gastrointestinal disorders as many studies have highlighted the psychophysical nature of gastrointestinal issues.

Several studies have shown that anxiety and stress play an important role in musculoskeletal diseases and reduce quality of life [25]. A previous study concluded that psychosocial stress and anxiety have an important role in increased depression and musculoskeletal diseases [26]. According to the current study, the majority of patients with musculoskeletal issues had moderate depression levels. However, 57.5% of the patients had moderate to extremely severe anxiety levels.

The current study had few limitations in the design. One of those limitations is the possibility of sampling bias because samples were randomly selected from a small population of patients reported to the Gampaha Wickramarachchi Ayurveda Teaching Hospital, Sri Lanka. Therefore, generalizing the results to all the patients who receive Ayurveda treatments in the entire country would be a limitation. In addition, family history of depression and stressful events was not taken into consideration. Therefore, further studies should be conducted to take these limitations into consideration in study design. Furthermore, the genetic and environmental factors that influence the stress and depression levels should also be taken into consideration. Finally, the selection of sample size from different disorders was performed based on the number of admissions during the study period as inward patients. Therefore, samples' sizes in all categories were not the same. Therefore, larger sample size should also be considered in future studies.

In general, the majority of the patients were characterized by mild or moderate levels of stress in all the study groups when compared with the control population. This study evidenced the clinically important associations between psychiatric illness and chronic medical conditions. Therefore, it is a high priority to provide physical treatment along with the psychiatric stimulation in order to improve the quality of the health service, which ultimately reduces the time taken for recovery from the disorder condition. A better knowledge on the psychological morbidity would be beneficial to plan appropriate multidisciplinary approach to the care of patients. Therefore, the clinicians at Ayurvedic setup could be encouraged to look for these manifestations with more focus and consider in liaison with psychiatric services when necessary. Further, this may enhance the positive thinking among patients which indirectly improve the quality of life.

## 5. Conclusion

According to the results, high levels of distress present among the majority of the patients. The highest stress and anxiety levels were denoted from patient suffered from integumentary system-related issues, while nervous system-related issues denoted the highest anxiety. Therefore, it has become a dire need of doping a biopsychosocial approach to patient assessment and patient care, in actual practice. Hence, it will be useful in enhancing the treatment and patient care along with the psychological counselling and enhancing the quality of life.

## Figures and Tables

**Table 1:** Demographic and socioeconomic characterization patients with different disorders.

Variable	Category	Percentage of study populations					*X* ^*2*^ *p* value
		Control	Nervous system (*n*=50)	Musculoskeletal system (*n*=40)	Integumentary system (*n*=30)	Gastrointestinal system (*n*=28)	
*Age*	18–35 years	30.0	16.0	0	33.3	25.0	*X* ^*2*^ = 18.91 Df = 8 *p*=0.015^*∗*^
	35–65 years	50.0	52.0	62.5	50.0	50.0	
	>65 years	20.0	32.0	37.5	16.7	25.0	

*Gender*	Female	56.0	40.0	55.0	66.7	75.0	*X* ^*2*^ = 10.70 Df = 4 *p*=0.030^*∗*^
	Male	44.0	60.0	45.0	33.3	25.0	

*Locality*	Rural	22.0	12.0	42.5	33.3	25.0	*X* ^*2*^ = 23.07 Df = 8 *p*=0.003^*∗*^
	Semiurban	30.0	52.0	42.5	23.3	50.0	
	Urban	48.0	36.0	15.0	43.4	25.0	

*Marital status*	Clergy	4.0	2.0	5.0	16.7	3.6	*X* ^*2*^ = 28.08 Df = 12 *p*=0.005^*∗*^
	Single	20.0	12.0	5.0	33.3	25.0	
	Married	62.0	74.0	77.5	50.0	53.6	
	Divorced/widowed	14.0	12.0	12.5	0	17.9	

*Family status*	Nuclear	20.0	36.0	30.0	50.0	17.9	*X* ^*2*^ = 31.096 Df = 12 *p*=0.006^*∗*^
	Extended	30.0	46.0	45.0	16.7	14.3	
	Married without children	34.0	4.0	7.5	0	35.7	
	Alone or other	16.0	14.0	17.5	33.3	32.1	

*Ethnicity*	Sinhalese	46.0	98.0	95.0	50.0	53.6	*X* ^*2*^ = 66.34 Df = 12 *p*=0.001^*∗*^
	Tamil	28.0	0	0	20.0	28.6	
	Muslim	16.0	2.0	5.0	20.0	0	
	Burgher	10.0	0	0	10.0	17.8	

*Religion*	Buddhist	40.0	80.0	85.0	50.0	53.6	*X* ^*2*^ = 46.76 Df = 12 *p*=0.004^*∗*^
	Christian	20.0	18.0	10.0	16.7	25.0	
	Hindu	24.0	0	0	13.3	21.4	
	Islam	16.0	2.0	5.0	20.0	0	

*Occupation*	Government servant	24.0	26.0	22.5	0	28.6	*X* ^*2*^ = 60.91 Df = 28 *p*=0.0001^*∗*^
	Army/forces	6.0	2.0	0	16.7	0	
	Semigovernment	14.0	2.0	0	0	0	
	Private company	12.0	10.0	10.0	0	21.4	
	Self-employed	2.0	6.0	15.0	16.7	7.1	
	Labourer/farmer	8.0	12.0	7.5	16.7	21.4	
	Traders/business	6.0	12.0	12.5	16.7	7.1	
	None	28.0	30.0	32.5	33.3	14.3	

*Social status*	Low	30.0	60.0	22.5	16.7	25.0	*X* ^*2*^ = 37.85 Df = 8 *p*=0.001^*∗*^
	Medium	48.0	26.0	77.5	66.6	50.0	
	High	22.0	14.0	0	16.7	25.0	

*Educational level*	Primary	32.0	42.0	47.5	50.0	50.0	*X* ^*2*^ = 12.82 Df = 8 *p*=0.118
	Secondary	56.0	50.0	45.0	33.3	25.0	
	Tertiary	12.0	8.0	7.5	16.7	25.0	

*Monthly income in Sri Lankan rupee*	<5,000	2.0	12.0	17.5	16.7	10.7	*X* ^*2*^ = 41.14 Df = 16 *p*=0.001^*∗*^
	5,001–10,000	4.0	6.0	10.0	0	0	
	10,001–20,000	16.0	20.0	15.0	0	25.0	
	20,001–30,000	48.0	18.0	20.0	66.7	28.6	
	>35,000	30.0	44.0	37.5	16.7	35.7	

*Note*. All *X*^*2*^ and *p* values are based on “chi-square test for independence” analysis of numbers of patients or respondents. The asterisk (^*∗*^) over the *p* value indicates significant dissimilarities among the study populations (*p* < 0.05 at 95% level of significance).

**Table 2:** Mean scores for stress, anxiety, and depression relevant to study population.

Study group	Stress	Anxiety	Depression
Control population	8.90 ± 0.78^a^	7.30 ± 0.58^a^	9.50 ± 1.08^a^
	(8.12 ± 9.68)	(6.72 ± 7.88)	(8.42 ± 10.58)

Nervous system	24.0 ± 1.47^b^	18.6 ± 1.51^b^	23.2 ± 1.78^b^
	(22.53 ± 25.47)	(17.09 ± 20.11)	(21.42 ± 24.98)

Musculoskeletal system	20.6 ± 1.61^b^	12.6 ± 1.51^c^	16.8 ± 1.57^b^
	(18.99 ± 22.21)	(11.09 ± 14.11)	(15.23 ± 18.37)

Integumentary system	27.7 ± 2.54^b^	6.0 ± 2.73^a^	31.7 ± 3.42^c^
	(25.16 ± 30.24)	(3.27 ± 8.73)	(28.28 ± 35.12)

Gastrointestinal system	25.0 ± 2.65^b^	16.0 ± 2.42^b^	19.5 ± 3.77^b^
	(22.35 ± 27.65)	(13.58 ± 18.42)	(15.73 ± 23.27)

*p* value	**0.04**	**0.03**	**0.12**

*Note*. Different superscript letters in a column indicate groups with significant differences (*p* < 0.05) at 0.95% level of confidence.

## Data Availability

The data used to support this study are included within the article and are available upon request to the corresponding author via mail.

## References

[1] https://www.who.int/governance/eb/who_constitution_en.pdf

[2] Mushtaq M., Zahir M. (2016). Depression, anxiety, stress and other effect upon the self-efficacy in dengue patients. *Journal of Postgraduate Medical Institute*.

[3] Dash B. (2005). *Fundamentals of Ayurveda Medicine*.

[4] Krejcie R. V., Morgan D. W. (1970). Determining sample size for research activities. *Educational and Psychological Measurement*.

[5] Osman A., Wong J. L., Bagge C. L., Freedenthal S., Gutierrez P. M., Lozano G. (2012). The depression anxiety stress scales-21 (DASS-21): further examination of dimensions, scale reliability, and correlates. *Journal of Clinical Psychology*.

[6] Hollifield M., Hewage C., Gunawardena C. N., Kodituwakku P., Bopagoda K., Weerarathnege K. (2008). Symptoms and coping in Sri Lanka 20-21 months after the 2004 tsunami. *The British Journal of Psychiatry*.

[7] Williams S. S., Wijesinghe C. A., Jayamanne S. F. (2011). Delayed psychological morbidity associated with snake bite envenoming. *PLoS Neglected Tropical Diseases*.

[8] Rekha A. Adaptation and validation of the depression anxiety and stress scale (DASS21) among students in the university of Colombo.

[9] Chandradasa M., Kuruppuarachchi K. A. L. A. (2017). Child and youth mental health in post-war Sri Lanka. *BJPsych. International*.

[10] Chandradasa M., Champika L., Gunathillaka K., Mendis J. (2016). Association of duration of untreated psychosis and functional level, in first episode of schizophrenia attending an outpatient clinic in Sri Lanka. *Journal of the Postgraduate Institute of Medicine*.

[11] Jhanjee A., Bhatia M. S., Srivastava S., Rathi A. (2013). A study of psychiatric symptomatology in dengue patients. *Delhi Psychiatry Journal*.

[12] Rodin G., Voshart K. (1986). Depression in the medically ill: an overview. *The American Journal of Psychiatry*.

[13] Bhatia M. S., Gautam P., Jhanjee A. (2015). Psychiatric morbidity in patients with chikungunya fever: first report from India. *Journal of Clinical and Diagnostic Research*.

[14] Cohen A. D., Ofek–Shlomai A. e., Vardy D. A., Weiner Z., Shvartzman P. (2006). Depression in dermatological patients identified by the mini international neuropsychiatric interview questionnaire. *Journal of the American Academy of Dermatology*.

[15] AlGhamdi K. M. (2010). Beliefs and perceptions of Arab vitiligo patients regarding their condition. *International Journal of Dermatology*.

[16] Picardi A., Abeni D., Melchi C. F., Puddu P., Pasquini P. (2000). Psychiatric morbidity in dermatological outpatients: an issue to be recognized. *British Journal of Dermatology*.

[17] Levy R. L., Olden K. W., Naliboff B. D. (2006). Psychosocial aspects of the functional gastrointestinal disorders. *Gastroenterology*.

[18] Locke G. R., Weaver A. L., Melton J., Talley N. J. (2004). Psychosocial factors are linked to functional gastrointestinal disorders: a population based nested case-control study. *The American Journal of Gastroenterology*.

[19] Wu J. C. (2012). Psychological co-morbidity in functional gastrointestinal disorders: epidemiology, mechanisms and management. *Journal of Neurogastroenterology and Motility*.

[20] Lydiard R. B., Fossey M. D., Marsh W., Ballenger J. C. (1993). Prevalence of psychiatric disorders in patients with irritable bowel syndrome. *Psychosomatics*.

[21] Oudenhove L. V., Vandenberghe J., Demyttenaere K., Tack J. (2010). Psychosocial factors, psychiatric illness and functional gastrointestinal disorders: a historical perspective. *Digestion*.

[22] Tse A. W. Y., Lai L. H., Lee C. C. (2010). Validation of self-administrated questionnaire for psychiatric disorders in patients with functional dyspepsia. *Journal of Neurogastroenterology and Motility*.

[23] Van O. L., Vandenberghe J., Geeraerts B. (2008). Determinants of symptoms in functional dyspepsia: gastric sensorimotor function, psychosocial factors or somatization?. *Gut*.

[24] Talley N. J., Fung L. H., Gilligan I. J., McNeil D., Piper D. W. (1986). Association of anxiety, neuroticism, and depression with dyspepsia of unknown cause. *Gastroenterology*.

[25] Li C.-Y., Chen K. R., Wu C. H., Sung F. C. (2001). Job stress and dissatisfaction in association with non-fatal injuries on the job in a cross-sectional sample of petrochemical workers. *Occupational Medicine*.

[26] Violante F. S., Fiori M., Fiorentini C. (2004). Associations of psychosocial and individual factors with three different categories of back disorder among nursing staff. *Journal of Occupational Health*.

